# Passive spatial sampling of mmWave and sub-THz propagation channels using omnidirectional lens antennas

**DOI:** 10.1038/s44459-026-00085-4

**Published:** 2026-09-23

**Authors:** Valentina Cicchetti, Andre Saker Andy, Theo Saunders, Jiahao Hu, Sana Salous, Yang Hao

**Affiliations:** 1 School of Electronic Engineering and Computer Science, Queen Mary University of London, London, UK; 2 Department of Engineering, Durham University, Durham, UK

**Keywords:** Engineering, Optics and photonics, Physics

## Abstract

6G wireless systems promise extreme data rates and dense spatial multiplexing, yet accurate characterization of propagation channels at mmWave and sub-THz frequencies remains challenging due to severe path loss and reliance on mechanically scanned directional antennas. Here, we demonstrate a wideband omnidirectional lens architecture enabling uniform near-horizon spatial sampling across the 75–100 GHz and 220–300 GHz bands. The design combines symmetric waveguide excitation, a conical field redistribution stage, and a dielectric lens to impose a transverse phase profile, producing stable azimuthal radiation with minimal angular bias. Functioning as a passive spatial sampling interface, the antenna captures multipath components across the full azimuth without mechanical scanning. Additively manufactured prototypes exhibit broadband impedance matching and realized gains of 7–8 dBi, with measurements matching full-wave simulations up to 300 GHz. Integrated into a wideband channel sounder in a SISO configuration, the platform enables the extraction of channel parameters in indoor environments, providing a scalable approach to channel characterization and future MIMO-relevant propagation modeling for 6G systems.

## Introduction

Sixth-generation (6G) wireless communications and high-resolution sensing systems increasingly rely on upper millimeter-wave (mmWave) and sub-terahertz (sub-THz) frequencies to enable multi-gigabit data rates, precise localization, and high-resolution imaging^1–3^. However, accurate characterization of propagation channels in these bands remains a fundamental challenge due to severe free space path loss, atmospheric absorption, and limited diffraction, compounded by the reliance on mechanically scanned directional antennas in existing measurement systems^4,5^. These limitations hinder efficient acquisition of spatial channel statistics and constrain the development of reliable models for emerging multiple-input multiple-output (MIMO) systems.

Omnidirectional antennas radiating near the horizon offer a promising approach for mmWave and sub-THz channel sounding. Unlike directional apertures that require mechanical rotation to reconstruct full 360° coverage, omnidirectional radiators inherently capture multipath components from all azimuth angles, enabling simultaneous spatial sampling of the propagation environment. This capability improves measurement efficiency, reduces calibration complexity, and preserves the natural angular structure of the channel. Previous studies have demonstrated rich multipath characteristics at sub-THz frequencies, including wide angular spreads and significant delay dispersion^6,7^, but have relied predominantly on mechanically scanned horn antennas, highlighting the need for compact broadband radiators capable of uniform azimuthal coverage.

Existing omnidirectional solutions at mmWave frequencies, such as open-ended waveguides, slotted cylinders, and biconical antennas, often suffer from limited bandwidth, pattern distortion, or complex assembly requirements^8,9^. More advanced designs, including TE_01_ circular-waveguide radiators^10,11^, extend operation to higher frequencies but typically require precision multi-layer fabrication. As a result, there remains a strong demand for omnidirectional antennas that combine broadband performance, compactness, and manufacturability for channel measurements and MIMO system evaluation. Importantly, full-azimuth reception enhances estimation of multipath components, spatial correlation, and angular diversity, which are critical parameters governing MIMO channel capacity^12,13^.

Dielectric lens antennas provide a promising platform for spatial field redistribution with high efficiency and compact form factor. By tailoring curvature and permittivity, lenses can transform the phase front of electromagnetic waves to achieve prescribed radiation characteristics. While classical Luneburg and axicon lenses, as well as recent gradient-index and metasurface implementations, have demonstrated broadband beamforming and high gain^14–22^, most designs focus on directive beams rather than the azimuthally uniform radiation required for omnidirectional channel sounding.

Additive manufacturing further expands the design space for mmWave and sub-THz antennas by enabling rapid realization of complex dielectric geometries. Stereolithography (SLA), with micrometer-scale resolution, is particularly well suited for such structures due to its smooth surface finish and controllable dielectric properties^23–25^. However, metallization remains a key challenge at these frequencies, where skin depths are on the order of micrometers and surface quality critically affects radiation efficiency. Various techniques, including conductive liquid paint (CLP), electroplating, and physical vapor deposition (PVD), present trade-offs between conductivity, uniformity, and cost^26–30^. Systematic evaluation of these approaches within a unified omnidirectional architecture remains limited.

In this work, we introduce a lens-based antenna architecture that enables passive spatial sampling of propagation channels across the 75–100 GHz and 220–300 GHz bands. The proposed design combines a rectangular-to-circular waveguide transition, a conical reflector for field redistribution, and a toroidal dielectric lens engineered to impose a controlled transverse phase profile, producing near-horizon omnidirectional radiation. Prototypes were fabricated using SLA additive manufacturing, and three metallization approaches, CLP, PVD, and computer numerical control (CNC) machined metal, were systematically evaluated. Experimental results show stable azimuthal radiation with gains of 7–8 dBi and excellent agreement with simulations up to 300 GHz. Integration into a wideband channel sounder further enables the extraction of large- and small-scale channel parameters, supporting MIMO-relevant propagation modeling in indoor sub-THz environments.

## Methods

### Antenna architecture and phase-synthesis method

This subsection describes the radiation objective, the radiating architecture, and the spherical–axicon toroidal lens synthesis used to realize near-horizon omnidirectional radiation. The design is motivated by sub-THz channel sounding, where uniform azimuthal sampling helps reduce angular bias in extracted large- and small-scale channel statistics.

The target radiation state is omnidirectional near the horizon, namely a uniform azimuthal response with peak radiation around *θ* ≈ 90° and limited variation (approximately ±2 dB) over *ϕ* ∈ [0°, 360°]. Two implementations are developed for the 75–100 GHz and 220–300 GHz bands. The antenna is designed to provide realized gains of 7 dBi to 8 dBi at band centers with less than 3 dB variation across each band, while maintaining elevation sidelobes below about −10 dB to limit spurious multipath capture. A compact geometry compatible with WR-10 and WR-3 waveguide interfaces is retained to support practical measurement deployments.

**Fig. 1:**
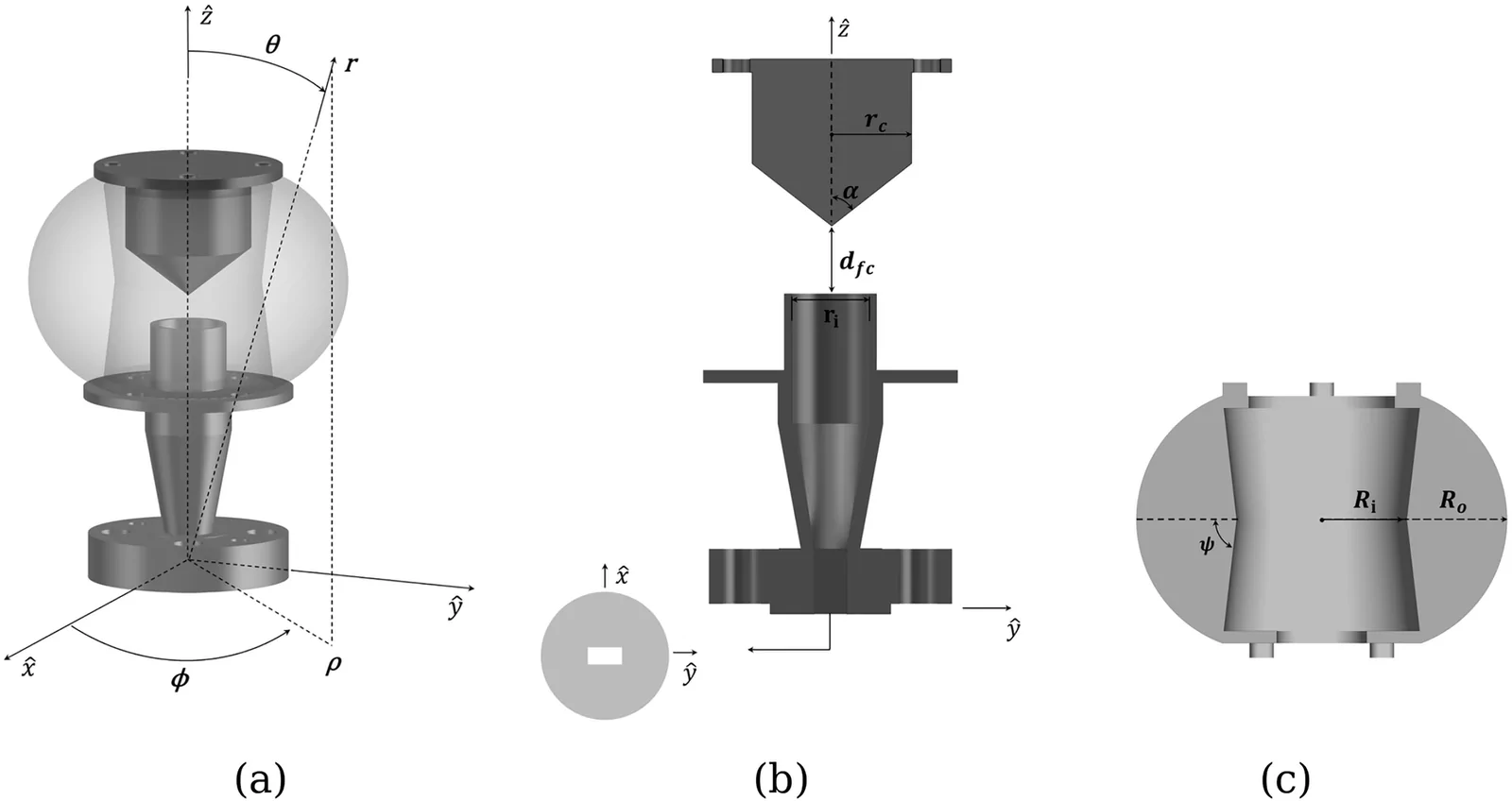
Geometry of the wideband omnidirectional spherical–axicon lens antenna. **a** 3D view, **b** cross-sectional feed and conical reflector, and **c** toroidal dielectric lens with spherical–axicon section.

The antenna comprises three electromagnetically coupled elements (Fig. 1). A broadband rectangular-to-circular transition converts the dominant TE_10_ mode of the waveguide input into a rotationally symmetric TE_11_ excitation at a circular aperture of radius *r*_*i*_, providing azimuthally uniform excitation for the subsequent shaping stages. A truncated conical metallic reflector, defined by semi-angle *α*, truncation radius *r*_*c*_, and axial spacing *d*_*fc*_, intercepts and redirects part of the radiated field outward, flattening the elevation response toward *θ* ≈ 90° and suppressing boresight radiation. A toroidal dielectric lens with a spherical–axicon cross-section, characterized by inner and outer radii (*R*_i_, *R*_o_), local thickness *t*(*ρ*), and axicon semi-angle *ψ*, imposes a tailored transverse phase delay that equalizes phase along a circular radiating rim and stabilizes azimuthal uniformity. Additional simulated field distributions supporting the design are provided in Supplementary Fig. S1.

Let *k*_0_ = 2*π*/*λ*_0_ be the free-space wavenumber and $n=\sqrt{{\varepsilon }_{r}}$ the refractive index of the lens material. The spatially varying phase delay imposed by the lens is modeled as

1 $$\Phi (\rho )={k}_{0}\left[n\,t(\rho )+{L}_{{\rm{air}}}(\rho )\right]+{\Phi }_{{\rm{ref}}}(\rho ),$$

where *t*(*ρ*) denotes the local lens thickness, *L*_air_(*ρ*) accounts for the residual air path from the effective feed phase center to the radiating rim, and *Φ*_ref_(*ρ*) represents the phase contribution associated with a single specular reflection on the conical surface. The synthesis objective is to enforce an *equal-phase* condition on a target radiating contour that supports near-horizon emission.

The target transverse phase is constructed by blending spherical and axicon terms. The spherical contribution

2 $${\Phi }_{{\rm{sph}}}(\rho )={k}_{0}\left(\sqrt{{\rho }^{2}+{z}_{{\rm{s}}}^{2}}-{z}_{{\rm{s}}}\right)$$

equalizes the optical path from an effective source at *z* = *z*_s_ to the aperture ring, while the axicon contribution

3 $${\Phi }_{{\rm{axi}}}(\rho )={k}_{0}\rho \sin \psi$$

introduces a linear radial phase ramp that promotes energy concentration toward large polar angles. The composite phase target is

4 $${\Phi }_{{\rm{des}}}(\rho )={\Phi }_{{\rm{sph}}}(\rho )+\beta \,{\Phi }_{{\rm{axi}}}(\rho )+{\Phi }_{0},$$

where *β* is a blending weight (typically 0.6–1.2) and *Φ*_0_ is an arbitrary constant. Combining (1) and (4) yields the thickness profile

5 $$t(\rho )=\frac{{\Phi }_{{\rm{des}}}(\rho )-{\Phi }_{{\rm{ref}}}(\rho )}{{k}_{0}\,n}-\frac{{L}_{{\rm{air}}}(\rho )}{n},$$

subject to manufacturability constraints $t(\rho )\ge {t}_{\min }$ and $t(\rho )\le {t}_{\max }$. In practice, *t*(*ρ*) is realized as a spherical cap smoothly blended with a conical phase ramp, forming a compact spherical–axicon toroidal section. The corresponding unwrapped phase distributions are shown in Supplementary Fig. S2.

The feed transition is designed to ensure azimuthally uniform TE_11_ excitation at the circular aperture. The radius is selected from the standard cutoff relation

6 $${r}_{i}=\frac{{X}_{11}^{{\prime} }}{2\pi }{\lambda }_{0},$$

where ${X}_{11}^{{\prime} }=1.841$ is the first root of ${J}_{1}^{{\prime} }(x)$, and the transition/circular lengths are optimized for low reflection and high modal purity following established mode-converter practice^31^. Across both bands, full-wave simulations predict return loss better than 15 dB, reaching below −20 dB near band centers.

**Fig. 2:**
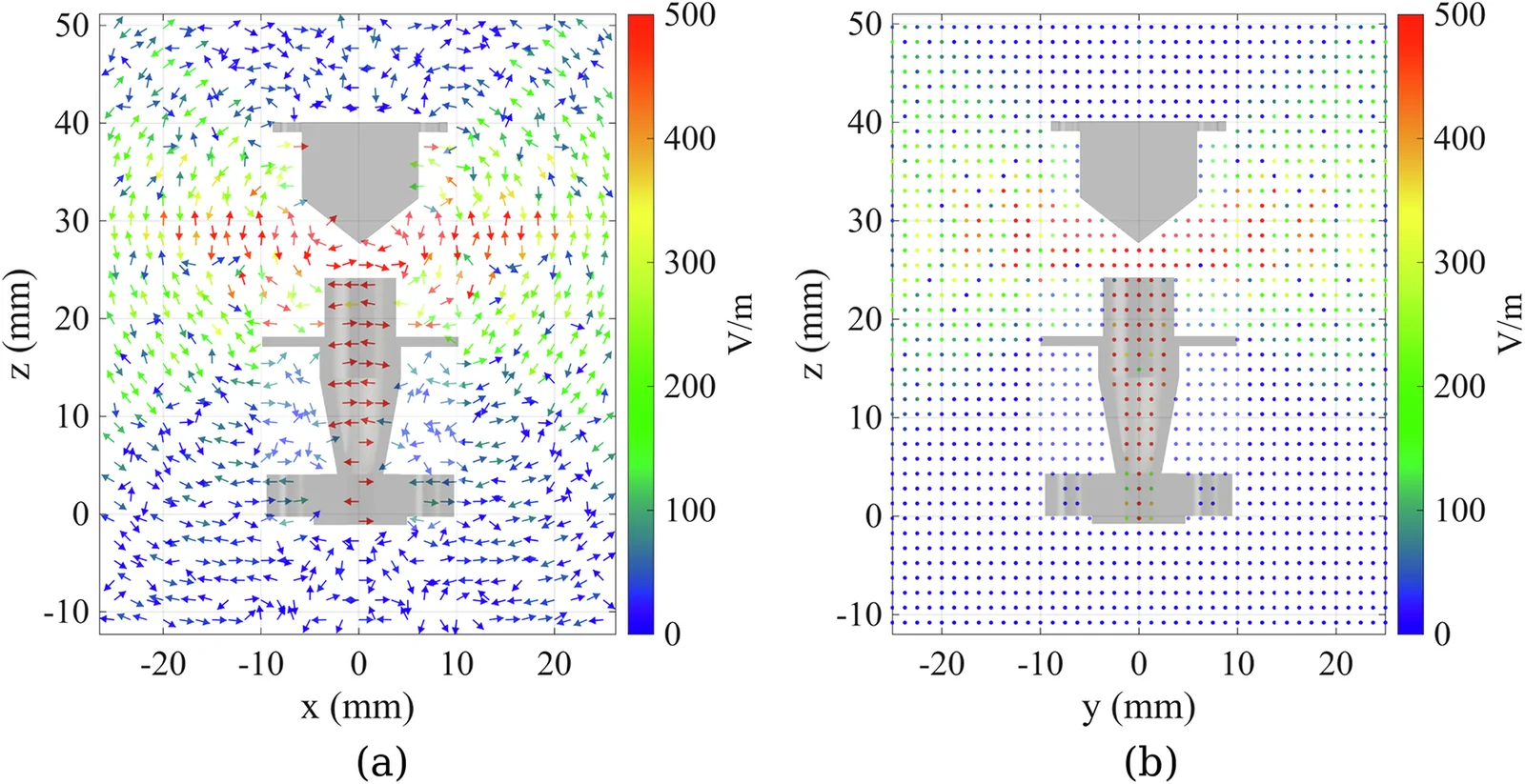
E-field redistribution by the conical reflector. Simulated E-field redistribution in the **a** *ϕ* = 0° plane and **b** *ϕ* = 90° plane at 90 GHz.

A coaxial conical reflector redirects energy toward the horizon and suppresses boresight radiation. Its semi-angle *α*, axial placement *d*_fc_, and truncation radius *r*_c_ are selected through ray-space preselection to steer specular rays into *θ* ∈ [70°, 110°] without blocking the direct path toward the lens rim, followed by full-wave refinement jointly with the lens profile *t*(*ρ*) to flatten the elevation pattern and reduce azimuthal ripple. The electric field distribution at 90 GHz in the principal planes (Fig. 2) confirms the intended redistribution prior to lens equalization. This behavior aligns with conic-section shaping approaches for omnidirectional pattern control^8,9^ and complements alternative mmWave/sub-THz implementations such as TE_01_ circular-waveguide radiators^10^.

**Fig. 3:**
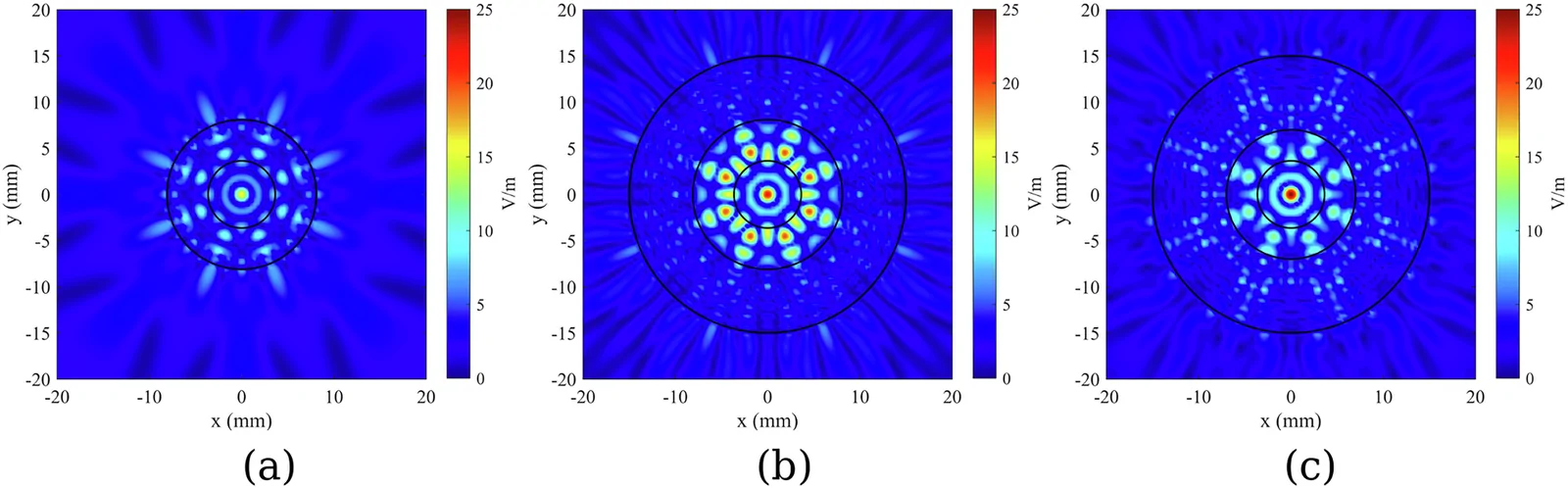
Simulated electric-field magnitude distributions for different lens profiles. Simulated electric-field magnitude distributions in the *x*–*y* plane at 100 GHz for **a** axicon, **b** hemispherical, and **c** spherical–axicon lenses. The hybrid design yields the most compact focal region and the highest on-axis field intensity.

The toroidal dielectric lens employs the spherical–axicon cross-section to equalize transverse phase and smooth the azimuthal distribution. The hybrid profile merges the focusing behavior of a spherical cap with the ring-focused behavior of an axicon, producing a compact focal region near the cone apex and improving near-horizon radiation. FEKO Altair Simulations at 100 GHz were conducted to compare different lens architectures using an omnidirectional source with propagation directions converging toward the lens center. The resulting simulated electric-field magnitude distributions in the *x*–*y* plane are presented in Fig. 3 with a half-lens truncation along the *z*-direction, clearly illustrating the distinct electromagnetic behaviors and the superior performance of the hybrid lens.

Specifically, the pure axicon lens (Fig. 3a) introduces a linear radial phase profile that spreads the electromagnetic energy over a wider radial zone, preventing a tight focus at the center. The hemispherical design (Fig. 3b) concentrates the field primarily near the structure but results in a broader, less defined focal spot with a significantly lower peak field intensity. Conversely, the composite spherical–axicon lens (Fig. 3c) achieves the most compact focal spot and the highest peak field intensity exactly at the central convergence point by successfully blending the localized focusing properties of the spherical curvature with the radial phase-ramping of the axicon profile. While this hybrid focusing approach has been previously demonstrated to enhance the performance of directive lens antennas^20,21^, the same physical principle can be successfully applied to shape and optimize the radiation characteristics of the proposed lens-equipped omnidirectional antenna.

To verify the validity of the phase synthesis in (1–5), full-wave simulations were carried out in CST Microwave Studio using complete 3D models of the feed–reflector–lens system. The final geometry was obtained through multiobjective optimization balancing reflector parameters (*α*, *d*_fc_, *r*_c_), lens radii (*R*_i_, *R*_o_), and phase-synthesis parameters (*ψ*, *β*). A complete model including mounting and metallization was simulated to verify azimuthal uniformity, cross-polarization suppression, and bandwidth stability.

Table 1 summarizes the final normalized geometrical parameters for the two designs (all distances normalized to *λ*_0_ at band center *f*_0_). The corresponding far-field patterns exhibit uniform azimuthal gain and low elevation ripple, supporting repeatable mmWave and sub-THz channel measurements. Material dispersion and loss were included using measured SLA resin properties: *ε*_*r*_ ≈ 2.75, $\tan \delta \approx 0.015$ in 75 GHz to 100 GHz, and *ε*_*r*_ ≈ 2.70, $\tan \delta \approx 0.025$ in 220 GHz to 300 GHz, consistent with printable photopolymer dielectric characteristics reported for mmW/THz applications^32,33^. Simulations indicate realized gains of 7 dBi to 8 dBi at band centers with less than 3 dB variation across each band, azimuthal ripple within approximately 2 dB, and elevation sidelobes below about −10 dB. These characteristics provide a compact, manufacturable solution for broadband omnidirectional near-horizon radiation in the 75 GHz to 300 GHz regime, supporting MIMO-relevant channel characterization where stable antenna patterns and calibrated angular coverage directly influence inferred channel statistics.

**Table 1:** Normalized geometrical parameters at band centers

Parameter	75–100 GHz	220–300 GHz
*R*_i_/*λ*_0_	2	2.4
*R*_o_/*λ*_0_	4.5	5.4
*ψ* (°)	83	83
*β*	0.9 to 1.2	0.9 to 1.2
*α* (°)	51.5	51
*d*_fc_/*λ*_0_	0.8	1.1

### Fabrication, metallization, and measurement procedures

Accurate mmWave and sub-THz electromagnetic modeling requires reliable dielectric properties for the additive manufactured parts. The dielectric constant and loss tangent of the white SLA resin used for the printed components were therefore characterized over both operational frequency ranges prior to antenna fabrication. The measurements were performed using a quasi-optical (QO) transmissometer^34–36^, enabling broadband extraction of complex permittivity from transmission (*S*_21_) data.

Square samples of 2.25 mm^2^ area and average thickness 1.3 mm were fabricated from the same resin batch used for the antenna prototypes. A Rohde & Schwarz ZNA vector network analyzer was connected to OML frequency extension modules to cover the W-band (75 GHz to 110 GHz) and J-band (220 GHz to 325 GHz). A four-mirror quasi-optical system was used to focus the transmitted field and reduce free-space spreading loss, with the sample placed at the focal plane between two phase-matched corrugated horn antennas.

**Fig. 4:**
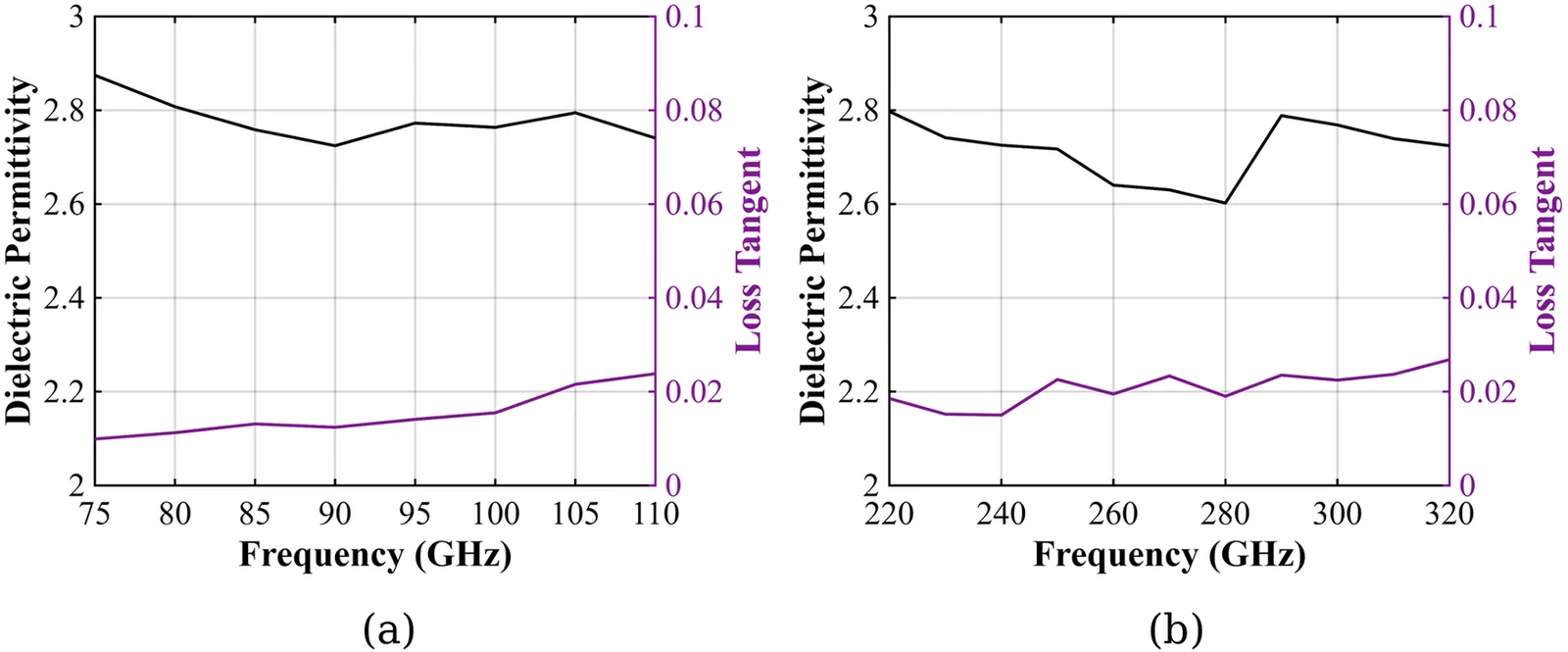
Measured dielectric properties of the SLA resin used for the 3D-printed lenses and feeds. **a** Permittivity and loss tangent in the W-band, 75–110 GHz. **b** Corresponding values in the J-band, 220–325 GHz.

The measured *S*_21_ data were converted to complex permittivity ${\varepsilon }_{r}=\varepsilon ^{\prime} -j\varepsilon ^{\prime\prime}$ using the ABCD-matrix method^37,38^. The dielectric constant was obtained from $\varepsilon {\prime}$, while the loss tangent was extracted from $\tan \delta =\varepsilon {\prime\prime} /\varepsilon {\prime}$. Figure 4 shows that the resin exhibits a stable permittivity of *ε*_*r*_ ≈ 2.75 in the W-band and *ε*_*r*_ ≈ 2.7 in the J-band, with a gradual increase of loss tangent from 0.01–0.02 across the W-band and up to ≈0.025 across the J-band. These values are consistent with low-loss SLA photoresins reported for mmWave sub-THz applications^32,33^ and were incorporated into all CST full-wave simulations. These losses lead to a difference of approximately 2 dB between the realized gain and directivity; nevertheless, this technique remains a cost-effective and precise solution.

**Fig. 5:**
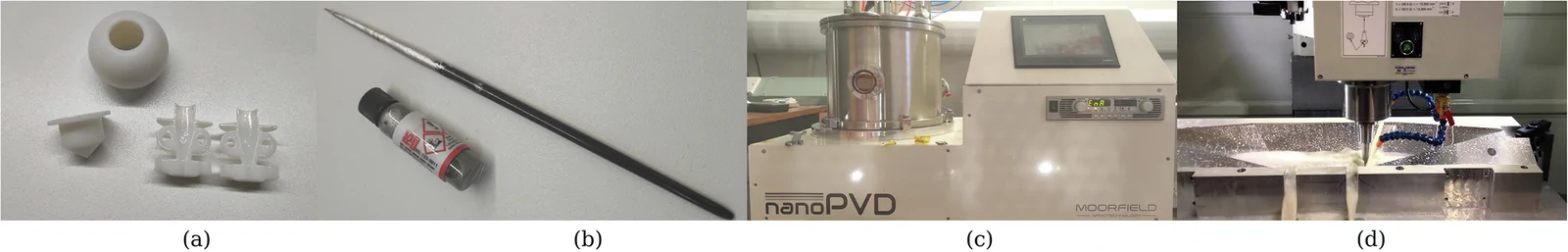
Workflow and metallization process for the proposed sub-THz lens antennas. **a** SLA printing and assembled W-band prototype, **b** CLP coating, **c** PVD sputtering, and **d** CNC machining of metallic parts.

Three fabrication routes, shown in Fig. 5, were explored to quantify the trade-offs between turnaround time, surface conductivity, and dimensional precision.

**Fig. 6:**
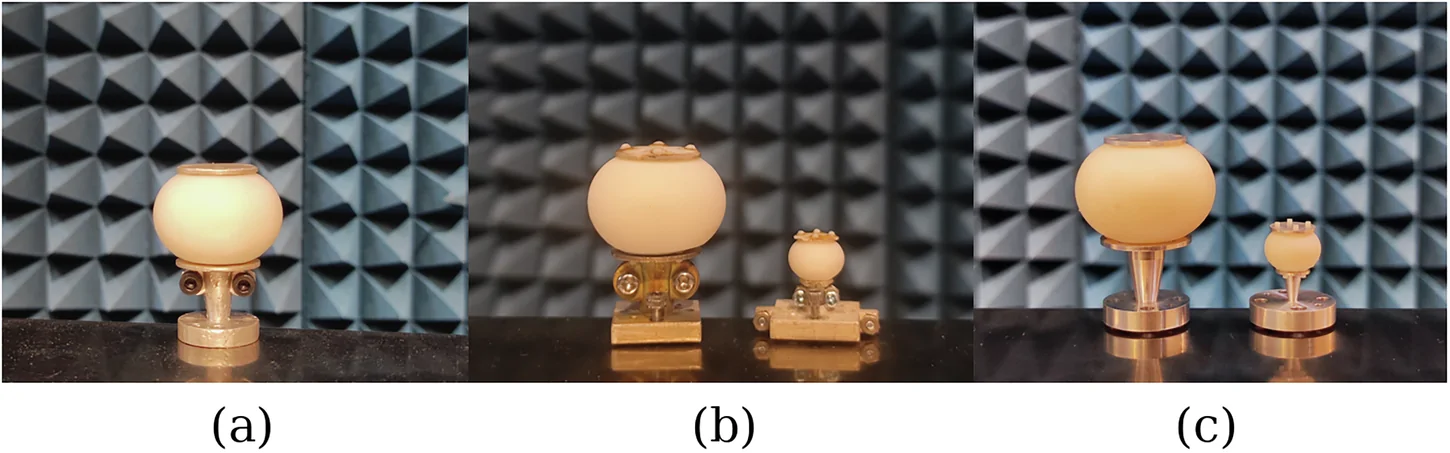
Fabricated prototypes using different metallization strategies. **a** CLP coating, **b** PVD sputtering, and **c** CNC machining.

In the first route, the feed transition, conical reflector, and dielectric lens were fabricated entirely by SLA 3D printing (Formlabs Form 3, 50 μm layer resolution) for the 75 GHz to 100 GHz bandwidth, with the resulting prototype shown in Fig. 6a. To facilitate metallization of internal surfaces, the feed was printed in two identical halves split along the longitudinal plane (*ϕ* = 0°) and reassembled using alignment holes and M3 screws. After cleaning with isopropyl alcohol and thermal curing, silver conductive liquid paint (RS Pro CLP) was applied manually in three uniform layers to maximize conductivity and adhesion. However, this process was not adopted for the 220 GHz to 300 GHz antenna. Indeed, as shown by successive results, radiation pattern distortions occur above 90 GHz, mainly due to low conductivity and surface roughness, which are critical parameters at these frequencies.

In the second route, the same SLA fabrication procedure was used, but metallization was performed by PVD sputtering to improve coating uniformity and conductivity, with the corresponding prototypes presented in Fig. 6b. This approach was applied to both prototypes, for 75 GHz to 100 GHz and 220 GHz to 300 GHz bands. A Moorfield NanoPVD 10S DC sputtering system deposited 99.9% silver under argon atmosphere at 2.8 × 10^−3^ mbar. Two deposition cycles with a 180° part rotation provided full coverage and yielded an average thickness of 1.17 μm (verified by four-point-probe measurements), exceeding the skin depth and enabling low-loss operation.

In the third route, the dielectric lens was printed by SLA, while the waveguide feed and conical reflector were CNC machined from aluminum 6082 to improve mechanical accuracy and repeatability, with the final assemblies shown in Fig. 6c. Precision machining achieved ±0.002 mm tolerance, and SURTEC 650 chromate-free conversion coating was applied to enhance corrosion resistance while preserving high conductivity. Minor adjustments near the rectangular-to-circular transition were introduced to simplify machining, with negligible impact on measured impedance or radiation behavior.

**Fig. 7:**
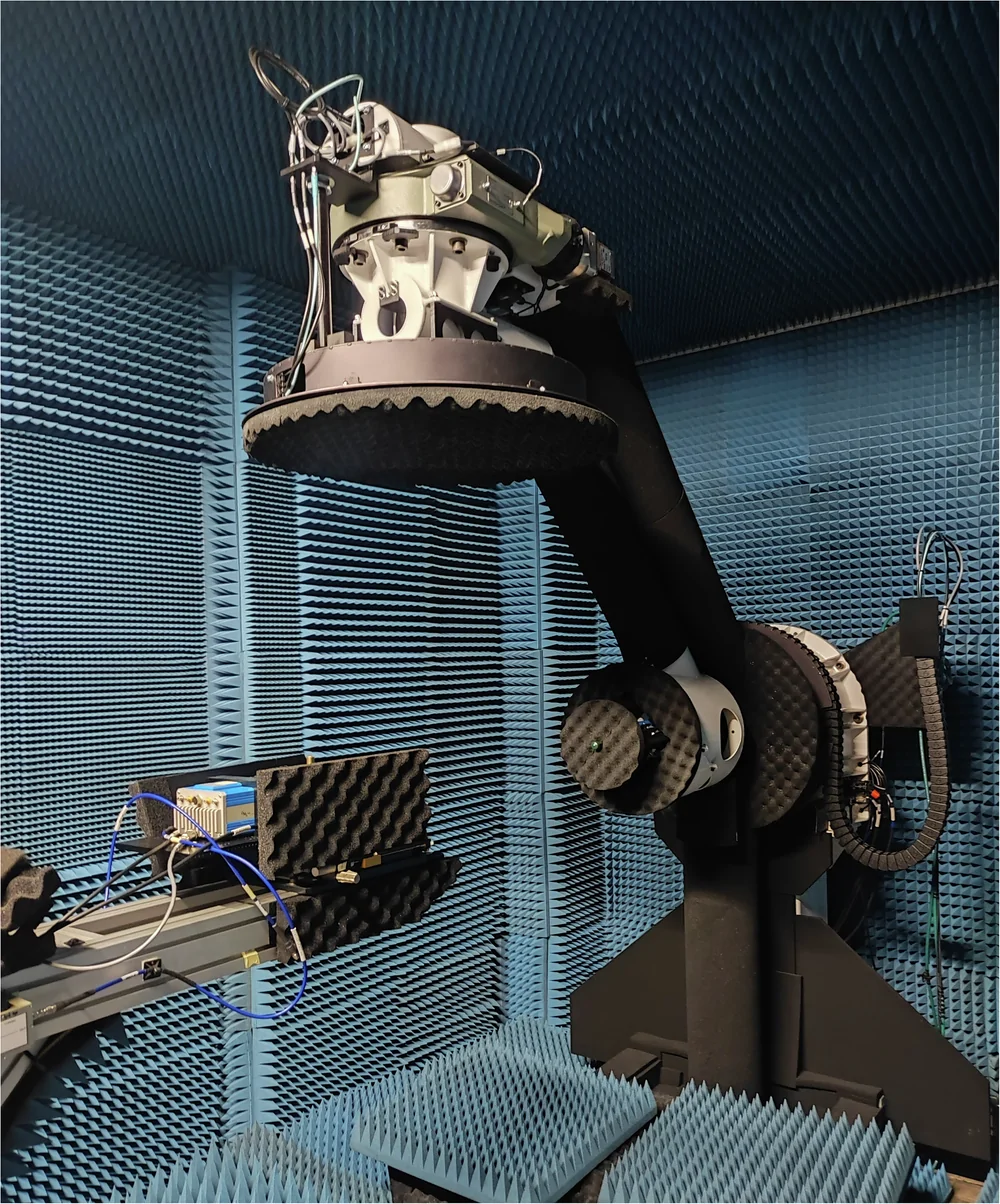
Spherical near-field antenna measurement setup. NSI-700S-360 spherical near-field antenna measurement setup used for characterizing the prototypes up to 500 GHz.

All prototypes were characterized in the spherical near-field antenna measurement facility (NSI-700S-360) at Queen Mary University of London, enabling accurate far-field reconstruction up to 500 GHz, as shown in Fig. 7. The antenna under test was mounted on a precision rotary stage while the probe scanned the radiated field over a spherical surface, and the far-field patterns were recovered by standard near-to-far transformation.

## Results

### Measured antenna performance

**Fig. 8:**
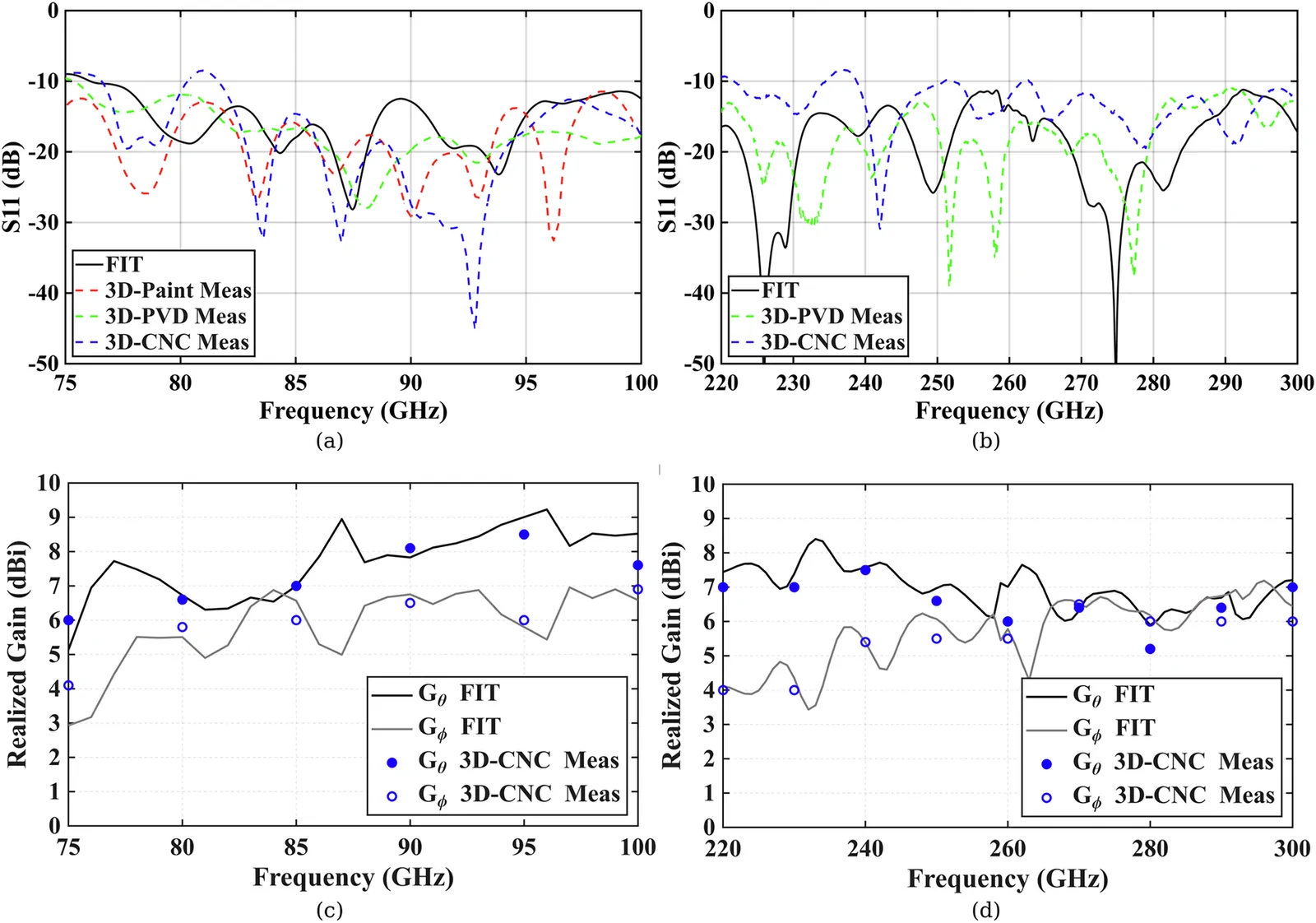
Simulated and measured antenna performance. **a** *S*_11_ for the 75–100 GHz prototype, **b** *S*_11_ for the 220–300 GHz prototype, **c** realized gain for the 75–100 GHz prototype, and **d** realized gain for the 220–300 GHz prototype.

Figure 8a, b compares measured and simulated input reflection coefficients for both bands, showing close agreement and validating the full-wave model and dielectric characterization. Minor deviations for the CNC prototypes at the lower band edges originate from small fabrication-driven adjustments in the transition geometry. Figure 8c, d shows the corresponding realized gain trends, which remain consistent across metallization routes and confirm that the spherical–axicon lens synthesis is robust to practical manufacturing variation. Maximum realized gains for the evaluated prototypes are summarized in Supplementary Table S1.

**Fig. 9:**
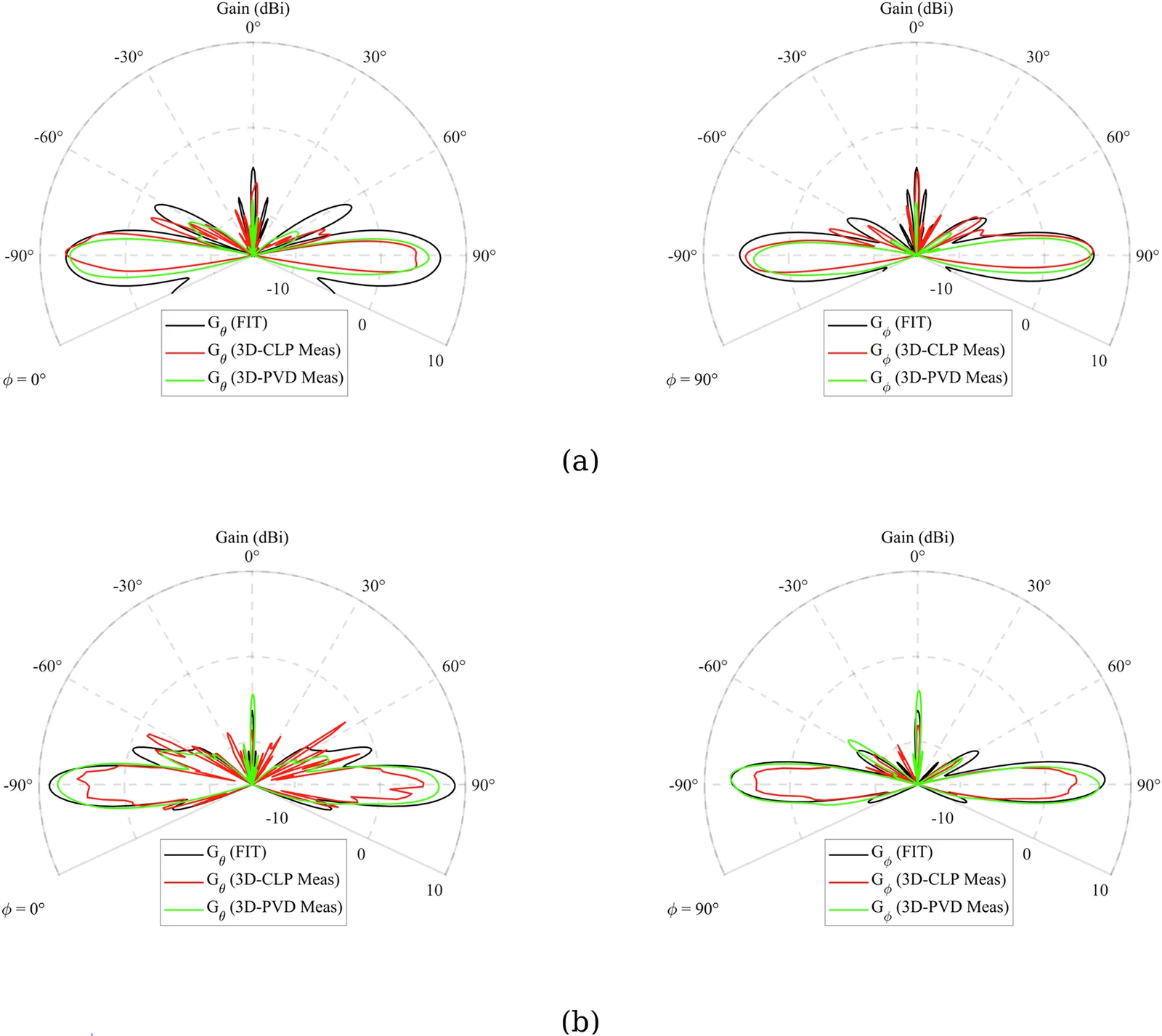
Radiation patterns for W-band prototypes metallized using CLP and PVD. Radiation patterns at **a** 80 GHz and **b** 100 GHz.

**Fig. 10:**
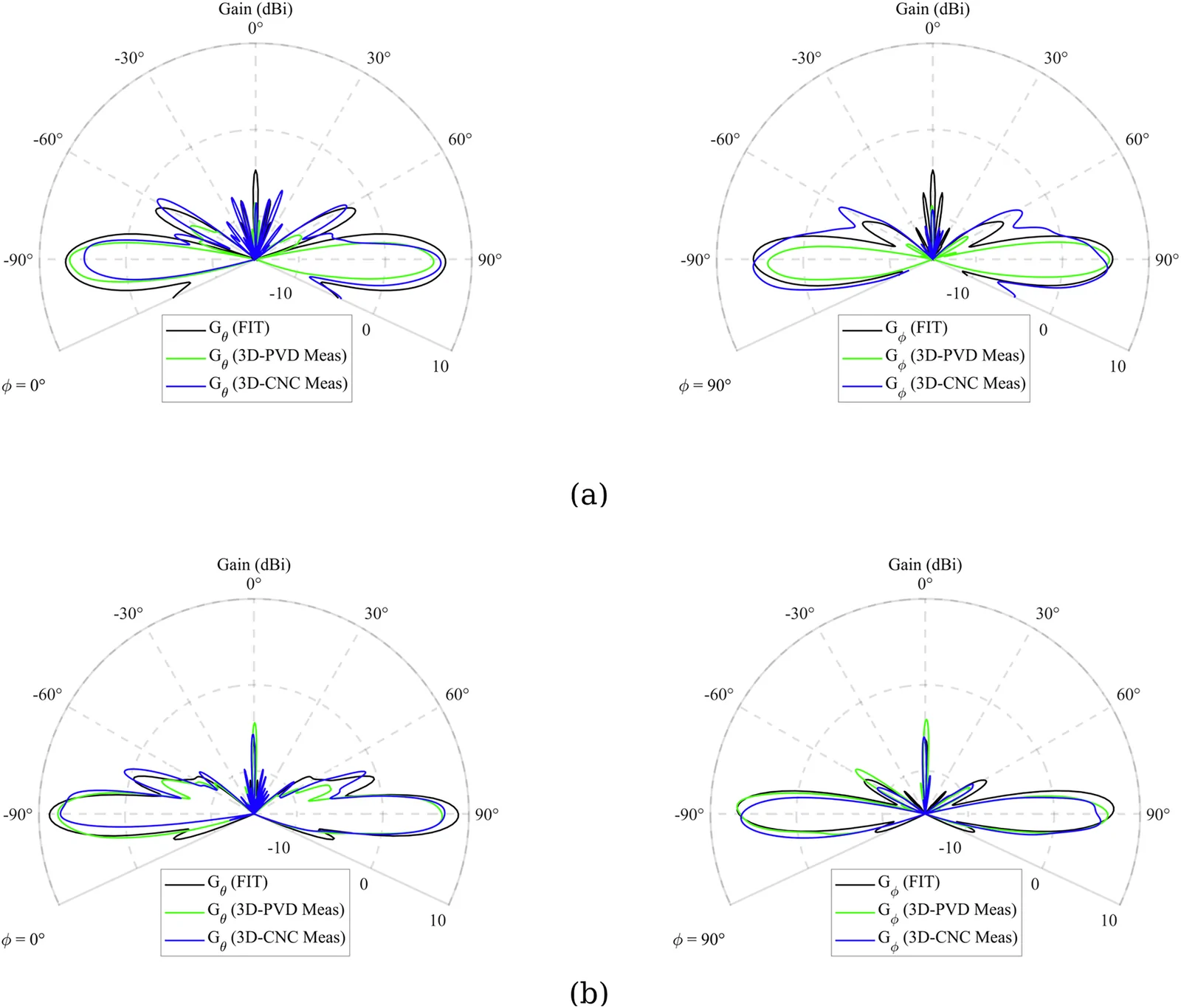
Radiation patterns for W-band prototypes metallized using PVD and CNC. Radiation patterns at **a** 80 GHz and **b** 100 GHz.

Radiation patterns were measured at representative frequencies to validate near-horizon omnidirectional behavior and to quantify the impact of metallization quality on azimuthal stability. In the W-band, the CLP and PVD prototypes exhibit comparable main-lobe shaping and near-horizon peaking (Fig. 9). The CLP-coated antenna shows increased ripple at higher frequencies, attributed to coating thickness non-uniformity and surface roughness, while the PVD-coated prototype maintains smoother patterns and improved gain stability at 100 GHz. A direct comparison between the PVD and CNC prototypes in W-band (Fig. 10) shows close agreement in main-beam shape and sidelobe levels, indicating that PVD can approach CNC performance when coating uniformity and assembly alignment are well controlled. Additional W-band radiation patterns at 90 GHz are provided in Supplementary Figs. S3 and S4.

**Fig. 11:**
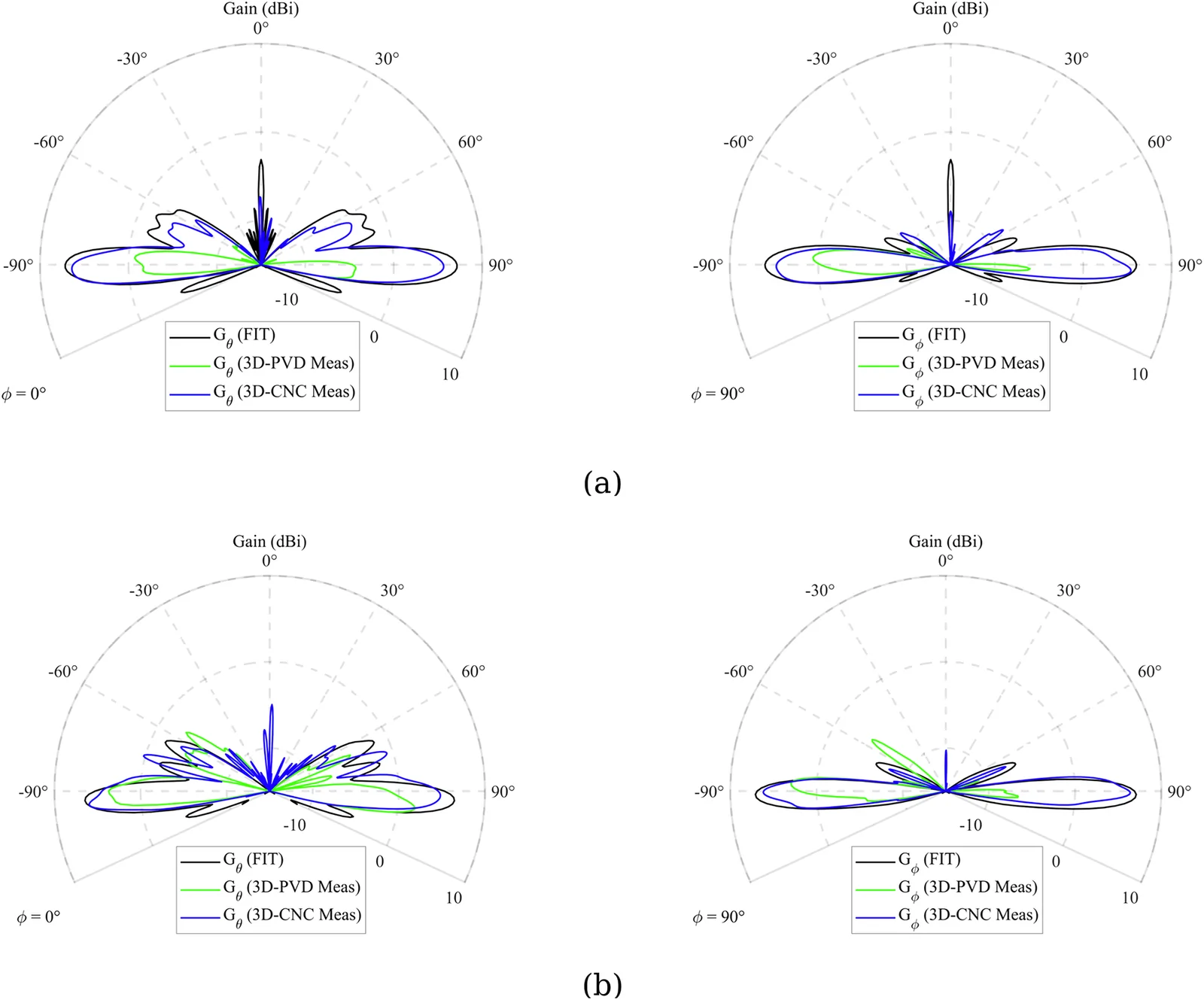
Radiation patterns for 220–300 GHz prototypes metallized using PVD and CNC. Radiation patterns at **a** 240 GHz and **b** 300 GHz.

In the 220–300 GHz band, the PVD and CNC prototypes were characterized at 240 GHz and 300 GHz (Fig. 11). At the upper end of the band, the PVD prototype exhibits more noticeable pattern deformation and reduced peak gain, consistent with increased sensitivity to metallization thickness, surface continuity, and alignment tolerances in the multi-part assembly. In contrast, the CNC-based prototype maintains stronger pattern symmetry and gains stability, confirming the benefit of improved mechanical precision and electrical continuity at sub-THz frequencies. These results confirm that the proposed architecture maintains azimuthal stability across fabrication methods and frequency bands. An additional J-band radiation pattern at 270 GHz is provided in Supplementary Fig. S5.

Overall, the three fabrication routes demonstrate that the proposed lens architecture is compatible with additive manufacturing while retaining measurement-grade behavior. CLP enables rapid, low-cost prototyping; PVD improves surface uniformity and conductivity when coating thickness and coverage are well controlled. CNC-assisted fabrication provides the highest mechanical precision and the most stable performance at sub-THz frequencies.

### Benchmark comparison with representative omnidirectional antennas

Table 2 summarizes the positioning of the proposed antenna relative to representative mmWave omnidirectional antennas reported in the literature.

**Table 2:** Comparison with representative mmWave and sub-THz omnidirectional antennas

Work	Band	Antenna concept	Gain	Main distinction from this work
Ma et al.^10^	Ka-band	TE_01_ circular-waveguide omnidirectional radiator	NR	Lower-frequency waveguide radiator; no validation in the 75–100 GHz or 220–300 GHz bands and no integration with a sub-THz channel sounder.
Verma et al.^11^	mmWave	Shaped circular-waveguide omnidirectional antenna	NR	Omnidirectional waveguide antenna, but without broadband W-band/J-band validation or channel-sounding demonstration.
Zhang et al.^9^	25.6–40.0 GHz	Biconical antenna with annular metal lens	9.2 dBi	High-gain Ka-band omnidirectional antenna; not demonstrated at 75–100 GHz or 220–300 GHz and not integrated into a sub-THz channel sounder.
Hussain and Kim^12^	5G mmWave	Integrated MIMO antenna module with 360° pattern diversity	>6 dBi	MIMO-oriented antenna module; not designed as a broadband sub-THz channel-sounding receive aperture.
This work	75–100 GHz; 220–300 GHz	Spherical–axicon omnidirectional lens antenna	7–8 dBi	Broadband mmWave/sub-THz omnidirectional lens antenna with additive/CNC-assisted fabrication and proof-of-concept integration into a 290-GHz channel-sounding experiment.

As shown in Table 2, previous omnidirectional antennas mainly target Ka-band or lower mmWave operation, high-gain communication coverage, or MIMO pattern diversity. In contrast, the present work combines broadband near-horizon omnidirectional radiation, manufacturable SLA/CNC-assisted implementation, experimental validation up to 300 GHz, and proof-of-concept use as a passive receive aperture in a 290-GHz channel-sounding campaign.

### Channel measurement efficiency and propagation results

Accurate large-scale channel characterization is a prerequisite for sub-THz system design. Above approximately 170 GHz, commercially available omnidirectional antennas are scarce, and most measurement campaigns in the 220 GHz to 300 GHz band rely on directional horn antennas. Such configurations require mechanical scanning or synthetic beamforming to emulate full-azimuth coverage. The omnidirectional lens antenna developed in this work eliminates this requirement, enabling direct and rapid channel characterization without mechanical rotation.

To quantify this measurement-efficiency advantage, the receiver-side measurement burden was estimated for the present 59-location campaign. In the implemented setup, the proposed omnidirectional lens antenna was used at the receiver, requiring only one receive-antenna orientation at each measurement location. In contrast, a directional receive antenna with an angular coverage comparable to the transmitter horn half-power beamwidth of approximately 36° would require about 360°/36° ≈ 10 azimuthal pointings per receiver location to emulate full-azimuth reception. For the 59 receiver locations considered here, this corresponds to 59 receiver-side measurements with the proposed omnidirectional antenna, compared with approximately 590 receiver-side pointings for a comparable directional receive antenna. If finer angular sampling were required, for example, 10° or 5° steps, the number of receiver-side pointings would increase to 2124 or 4248, respectively. This comparison highlights the practical reduction in mechanical scanning burden, acquisition overhead, and calibration complexity enabled by the proposed passive full-azimuth receive aperture.

**Fig. 12:**
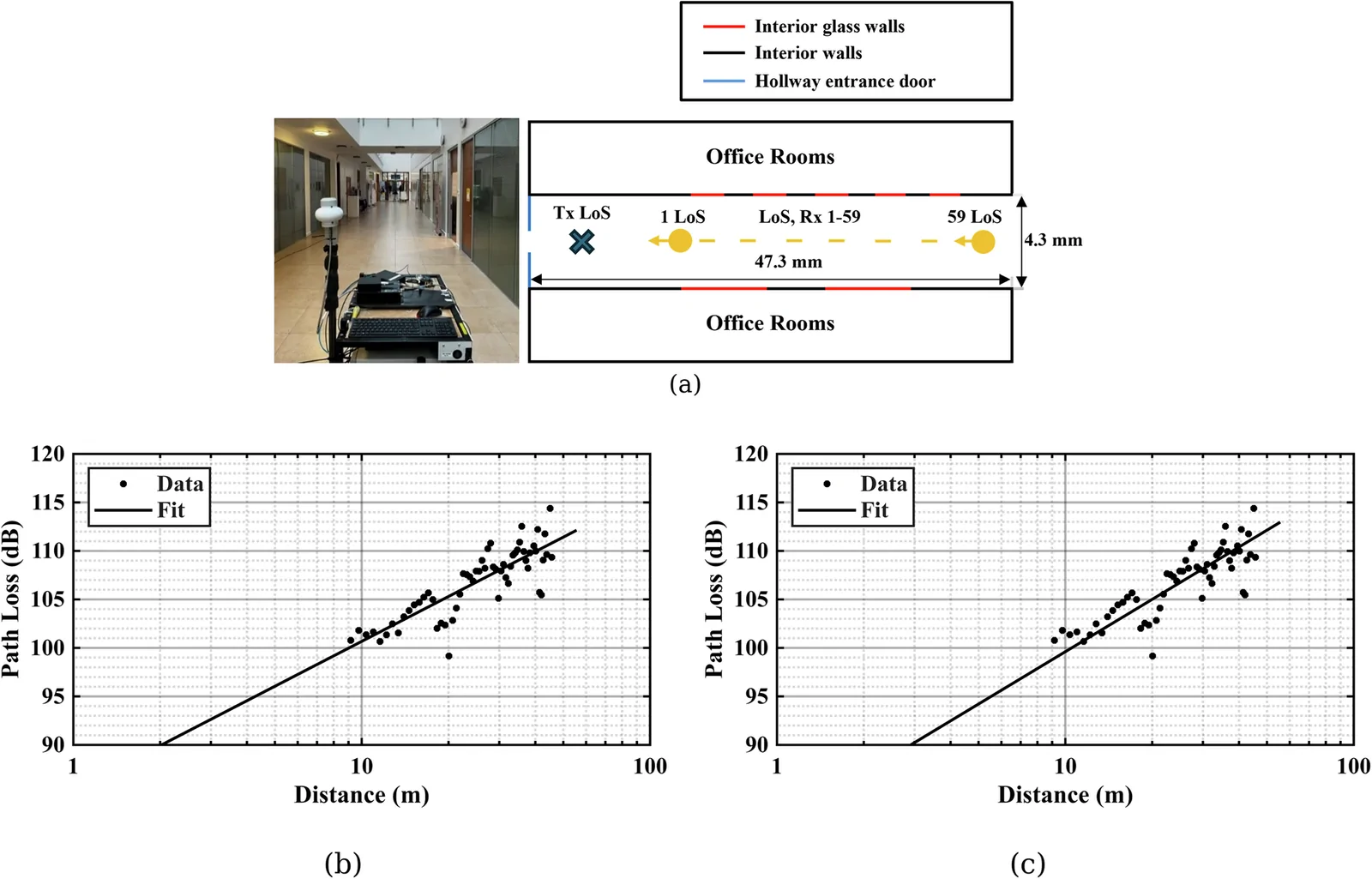
Path-loss measurement campaign in the 220–300 GHz band. **a** Hallway measurement environment and Tx–Rx geometry, **b** CI model fit, and **c** FI model fit.

To validate its practical utility, path-loss measurements were conducted in the 220 GHz to 300 GHz band using the wideband multi-frequency channel sounder developed at Durham University^4^. Measurements were performed in a hallway environment within the Palatine Centre building under line-of-sight (LoS) conditions. The transmitter (Tx) was positioned at a height of 2.9 m to simulate a typical access point, employing a standard horn antenna with an approximate 36 ° half-power beamwidth. Meanwhile, the receiver (Rx) was located at a height of 1.6 m to represent an average user, utilizing the omnidirectional lens antenna described in the “Methods.” The measurement geometry is shown in Fig. 12a.

Due to severe free-space attenuation and molecular absorption losses characteristic of the J-band, no valid signal was detected when the horn antenna was oriented −36° (counterclockwise) from the Line-of-Sight (LoS) direction. Consequently, measurements were restricted to the LoS (0°) and +36° (clockwise) orientations across 59 receiver locations, with transmitter-receiver (Tx-Rx) separation distances ranging from 9.2 to 45.6 m. At each position, data were recorded for a 1-s duration using a 2 GHz bandwidth centered at 290 GHz, utilizing an analog-to-digital converter (ADC) operating at a 100 MHz sampling rate.

It should be noted that the comparison in Table 3 concerns measurement burden rather than a direct experimental benchmark against a mechanically scanned directional receiver. The present study is a proof-of-concept SISO channel-sounding experiment using the proposed antenna as an omnidirectional receive aperture. The antenna captures the aggregate full-azimuth channel response, including the direct path and dominant reflected components, in a single receiver-side measurement. However, because no receiver-side angular scanning or antenna-array processing was performed, explicit angle-of-arrival extraction was not attempted in this work. Directional scanning remains necessary when high-resolution angular channel parameters are required.

**Table 3:** Estimated receiver-side measurement burden over 59 receiver locations

Receive approach	Azimuth sampling	Pointings per location	Total Rx pointings
Omnidirectional lens	Full-azimuth passive reception	1	59
Directional horn	36° HPBW	~10	~590
Directional scanning	10° step	36	2124
Directional scanning	5° step	72	4248

**Fig. 13:**
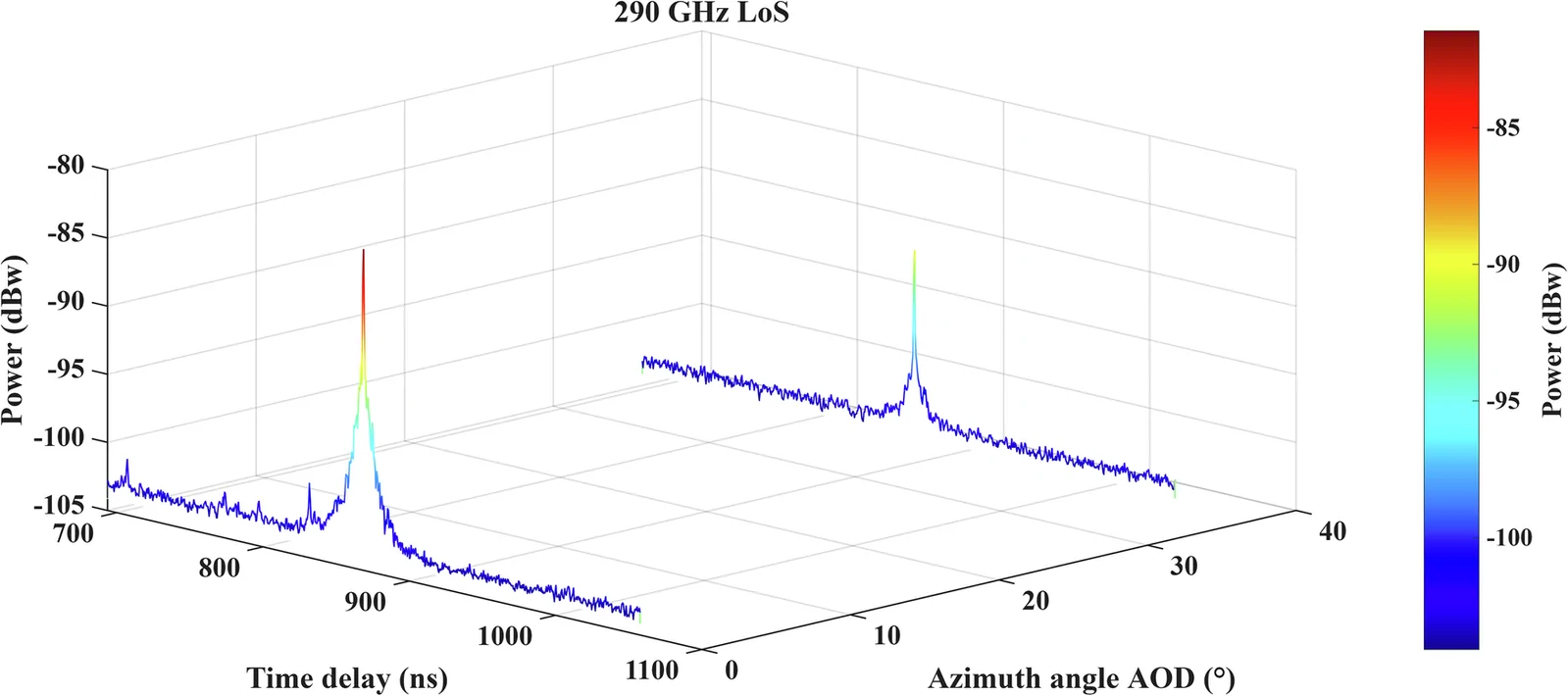
Computed power delay profile. Computed power delay profile at one measurement location for the two rotation angles of the transmit antenna. The receiving antenna was fabricated using the 3D-printing and CNC-machining technique at 290 GHz.

As illustrated in Fig. 13, representative power delay profiles (PDPs) were extracted to demonstrate the ability of the proposed omnidirectional receive antenna to capture temporally resolved multipath components. The plotted PDP corresponds to one measurement location and two transmitter orientations.

For the representative PDP, the RMS delay spread was calculated from the thresholded linear-power PDP. The PDP values were first converted from dB scale to linear power, and only delay bins exceeding the estimated median noise floor by 6 dB were retained. The RMS delay spread was then calculated as

7 $${\tau }_{{\rm{rms}}}=\sqrt{\frac{{\sum }_{i}{P}_{i}{({\tau }_{i}-\bar{\tau })}^{2}}{{\sum }_{i}{P}_{i}}},\quad \bar{\tau }=\frac{{\sum }_{i}{P}_{i}{\tau }_{i}}{{\sum }_{i}{P}_{i}},$$

where *P*_*i*_ is the linear power of the *i*-th retained delay bin and *τ*_*i*_ is the corresponding excess delay. Using this criterion, the representative RMS delay spreads are 2.84 ns and 0.33 ns for the 0° and 36° transmitter orientations, respectively, as summarized in Table 4.

**Table 4:** Representative PDP-derived temporal parameters at 290 GHz

Tx orientation	Threshold criterion	Retained bins	RMS-DS
0°	Median noise floor + 6 dB	32	2.84 ns
36°	Median noise floor + 6 dB	3	0.33 ns

These values are reported for the representative measurement location shown in Fig. 13; a full statistical RMS-delay-spread analysis over all 59 receiver locations would require processing the complete measurement dataset.

Following calibration, the path loss coefficients were estimated as shown in Fig. 12 using both the Close-In (CI) and Floating-Intercept (FI) formulations:

8 $${{\rm{PL}}}_{{\rm{CI}}}(d)={\rm{FSPL}}(1\,{\rm{m}})+10{n}_{{\rm{CI}}}{\log }_{10}\,\left(\frac{d}{1\,{\rm{m}}}\right)+{\chi }_{{\sigma }_{{\rm{CI}}}},$$

9 $${{\rm{PL}}}_{{\rm{FI}}}(d)=\alpha +10{n}_{{\rm{FI}}}{\log }_{10}(d)+{\chi }_{{\sigma }_{{\rm{FI}}}},$$

where *n* denotes the path-loss exponent, *α* the floating intercept, *χ*_*σ*_ the lognormal shadowing term, and *d* denotes the transmitter-receiver separation distance. Least-squares regression results are summarized in Table 5. The FI model provides a slightly improved statistical fit, while the CI model preserves a physically anchored 1 m reference.

**Table 5:** Estimated path-loss parameters at 290 GHz

Model	Intercept (dB)	Path-loss exponent *n*	*σ* (dB)
Close-in (CI)	82.1	1.78	1.95
Floating-intercept (FI)	85.3	1.54	1.89

## Discussion

The extracted exponents for both models sit below the free-space threshold, indicating indoor line-of-sight (LoS) waveguiding effects. Furthermore, the remarkably low shadow fading standard deviations (*σ*) underscore an excellent fit with minimal experimental noise^6,7^. As expected, the FI formulation yields a slightly lower variance due to its flexible intercept. These findings confirm the reliability of the omnidirectional lens antenna for accurate broadband sub-THz channel characterization. These parameters can inform stochastic or geometry-based channel models by providing measured path-loss and representative PDP-derived temporal information. Although the measurements are conducted in a SISO configuration, the omnidirectional receive aperture captures multipath contributions over the full azimuth, preserving the aggregate angular diversity of the propagation channel without the need for mechanical scanning. By combining PDP-derived temporal parameters with spatial sampling across multiple receiver locations, the approach provides a basis for future virtual-array or MIMO-oriented channel studies. However, explicit estimation of spatial correlation, effective rank, and angle-resolved multipath parameters will require additional receiver-side angular sampling, array measurements, or multi-antenna configurations.

In this sense, the proposed antenna operates as a passive spatial sampling interface, enabling scalable acquisition of channel measurements that are relevant to future MIMO-oriented studies at sub-THz frequencies.

This work has presented two broadband, low-cost, additively manufactured lens antennas operating in the 75 GHz to 100 GHz and 220 GHz to 300 GHz bands for mmWave and sub-THz communication and channel characterization. The proposed architecture, combining a rectangular-to-circular waveguide transition, a conical metallic reflector, and a toroidal dielectric lens with a spherical–axicon cross-section, enables near-horizon omnidirectional radiation with uniform azimuthal field distribution. This configuration provides measurement-grade spatial sampling without mechanical scanning, addressing a key limitation of sub-THz channel sounding above 170 GHz. This shifts channel sounding from mechanically scanned directional measurements to hardware-enabled spatial sampling.

Three fabrication strategies based on SLA additive manufacturing were evaluated, incorporating CLP, PVD, and CNC-machined metallic components. Spherical near-field measurements showed close agreement with full-wave simulations, validating the phase-synthesis design methodology and extracted material parameters. In the W-band, all prototypes achieved stable omnidirectional patterns with realized gains approaching 8 dBi. At higher frequencies, metallization quality became critical: CLP exhibited increased losses, PVD maintained good performance within coating limits, and CNC-based assemblies provided the highest mechanical precision and pattern stability, achieving peak gains of approximately 7 dBi in the 220 GHz to 300 GHz band.

Integration of the 220–300 GHz antenna into a wideband channel-sounding platform further demonstrated its practical utility. Indoor measurements yielded path-loss exponents and shadowing parameters consistent with prior sub-THz studies, confirming reliable link characterization. The omnidirectional receive aperture captures both direct and dominant reflected components without beam steering, enabling efficient extraction of large-scale attenuation and multipath statistics relevant to sub-THz MIMO channel modeling.

Overall, the results demonstrate that additive manufacturing, combined with controlled metallization, can deliver cost-effective and scalable mmWave and sub-THz antennas for measurement-driven system evaluation. The proposed lens architecture establishes a passive spatial sampling platform for broadband channel characterization, providing a practical pathway toward scalable MIMO channel acquisition, compact multi-antenna integration, and reconfigurable systems for emerging 6G communication and sensing applications.

## Supplementary information

Supplementary Information

## Data Availability

Simulation and measurement results used to produce the figures are provided with the manuscript submission. Additional data from this study are available from the corresponding author upon reasonable request.
