# Supplementary material for: Passive spatial sampling of mmWave and sub-THz propagation channels using omnidirectional lens antennas
Source: NPJ Wirel Technol. 2026 Sep 23;2(1):72. doi: 10.1038/s44459-026-00085-4 (PMC13600917; doi:10.1038/s44459-026-00085-4)
Supplement: Supplementary file 1 [file 44459_2026_85_MOESM1_ESM.pdf]

Supplementary Information

Passive Spatial Sampling of mmWave and Sub-THz Propagation Channels Using Omnidirectional Lens Antennas

This Supplementary Information file contains supplementary figures and one supplementary table that support the main manuscript. It does not contain a separate Supplementary Methods section; all methods required to reproduce the work are reported in the main manuscript.

Supplementary Figures

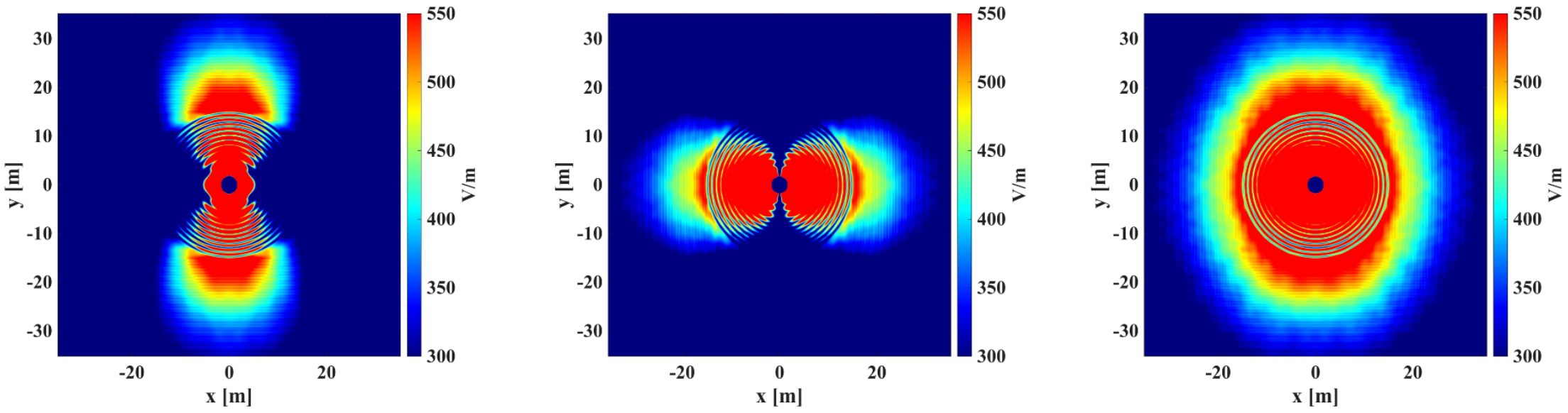

Figure S1. Electric-field distribution in the x–y plane at 100 GHz: (a) E_x, (b) E_z, and (c) $|E|$. The plots provide additional simulation evidence of the field redistribution and compact focal behaviour of the spherical–axicon lens profile.

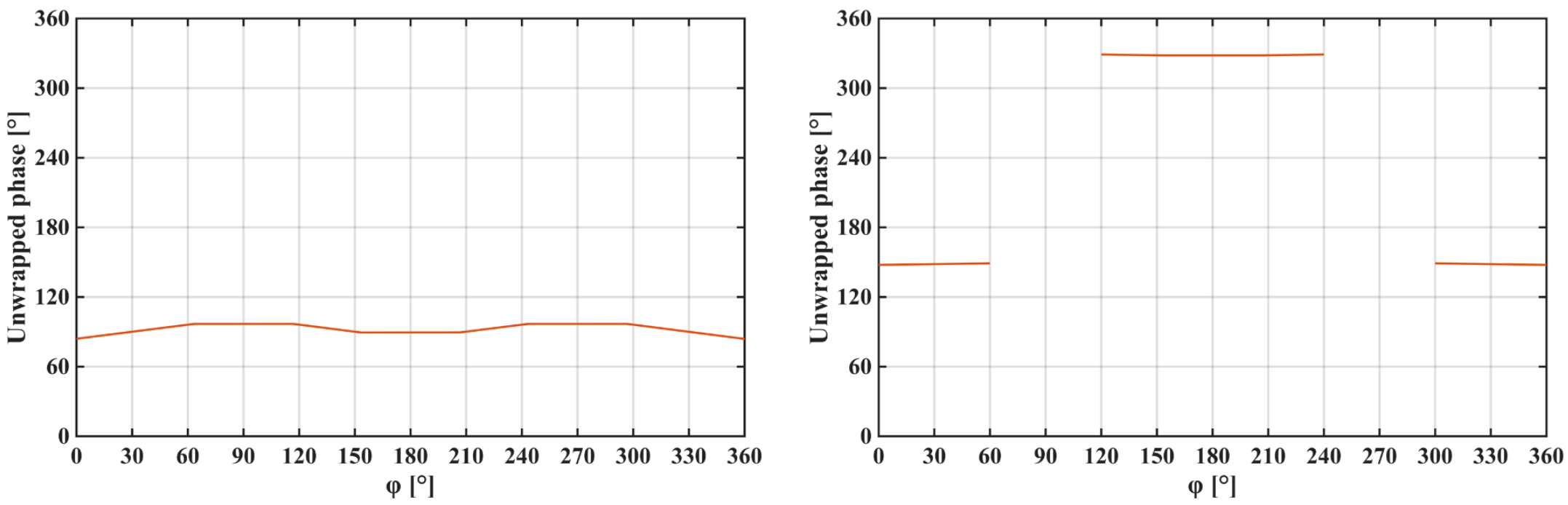

Figure S2. Unwrapped phase distribution of the electric field at 100 GHz: (a) E_x component and (b) E_z component, evaluated along a circular path close to the lens circumference.

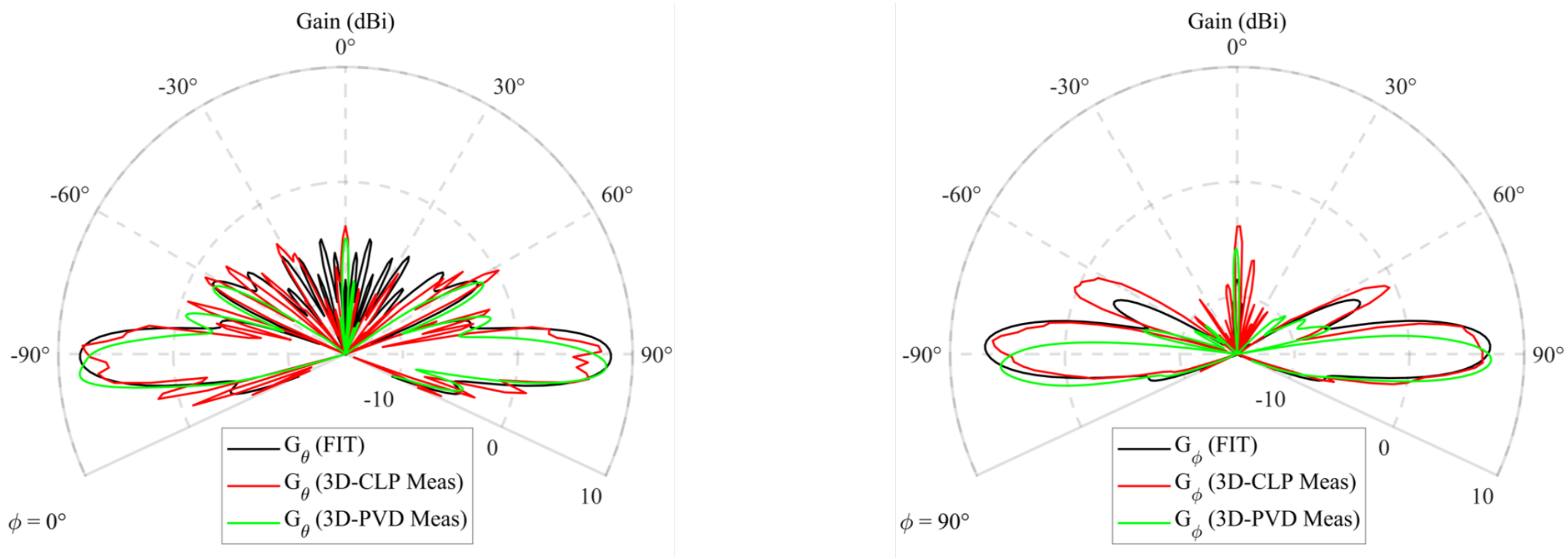

Figure S3. Measured radiation patterns of antennas fabricated using CLP and PVD metallisation at 90 GHz. The results provide an intermediate-frequency comparison within the W-band.

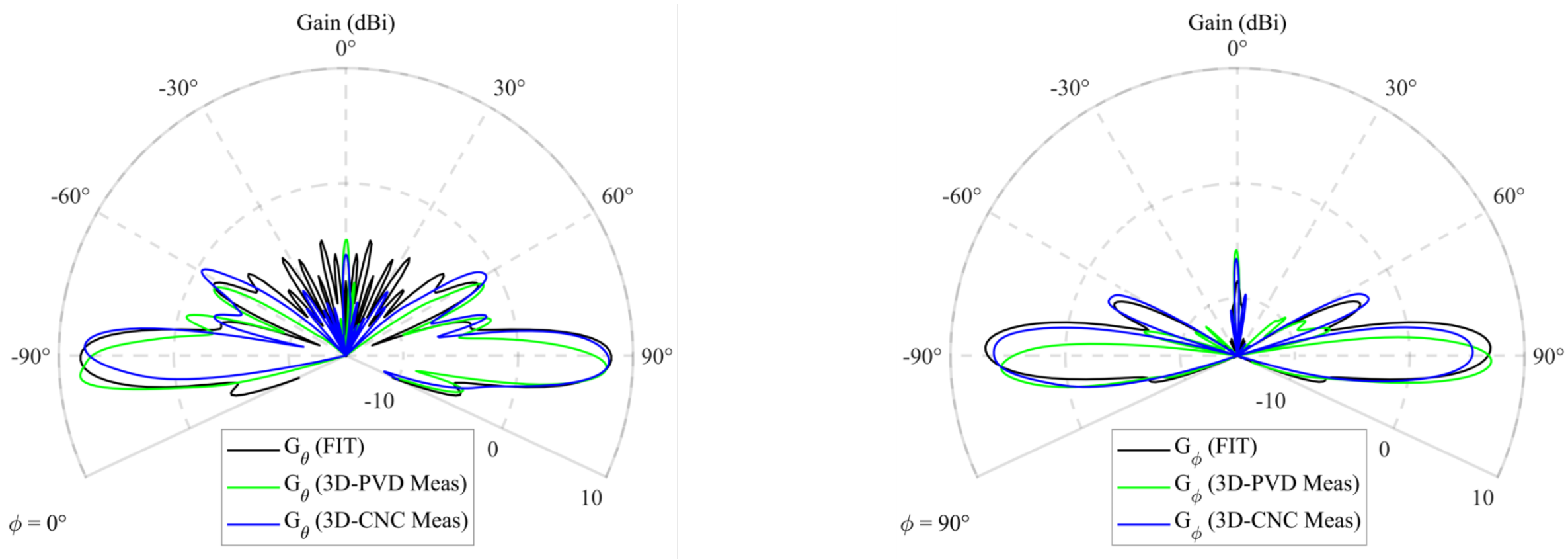

Figure S4. Measured radiation patterns of antennas fabricated using PVD and CNC approaches at 90 GHz. The results provide an intermediate-frequency comparison within the W-band.

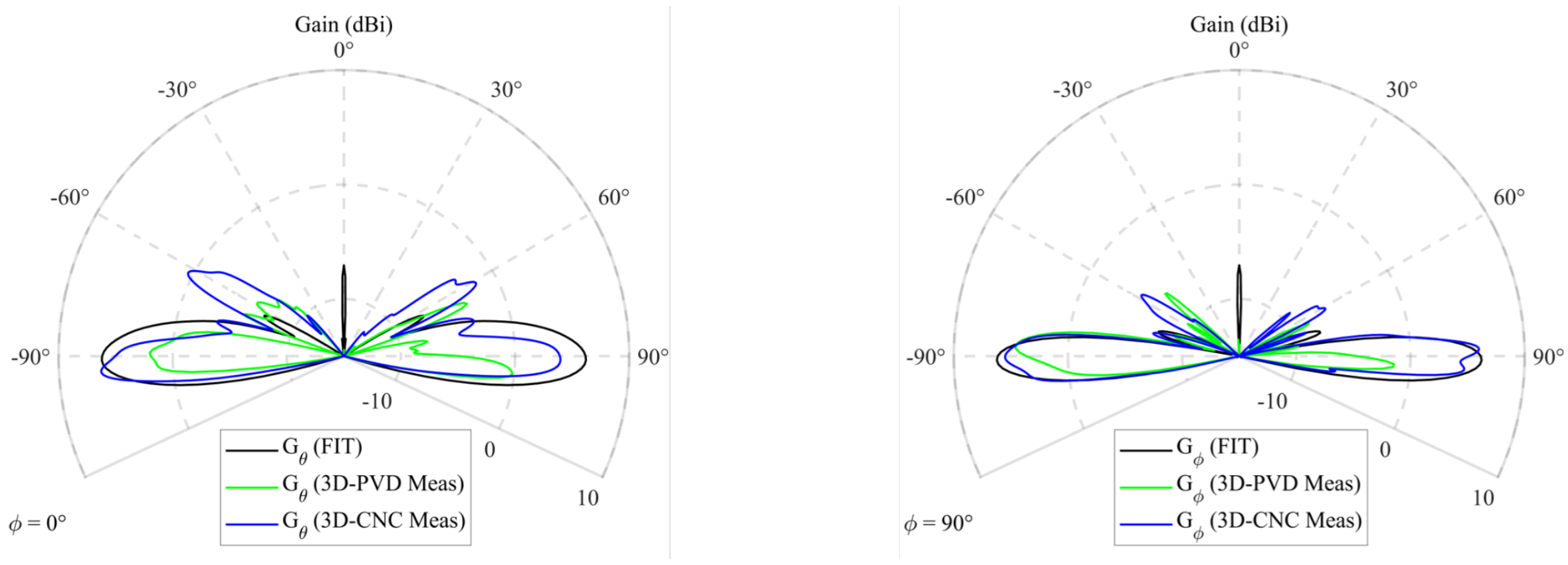

Figure S5. Measured radiation patterns of antennas fabricated using PVD and CNC approaches at 270 GHz. The results provide an intermediate-frequency comparison within the 220–300 GHz band.

Supplementary Table

Table S1. Maximum realised gain for the 75–100 GHz and 220–300 GHz antenna prototypes. A dash indicates that the corresponding prototype was not fabricated or not measured for that band.

Frequency	G_θ (dBi)				G_ϕ (dBi)			
(GHz)	FIT	CLP	PVD	CNC	FIT	CLP	PVD	CNC
**75–100 GHz band**								
80	7.0	7.0	6.7	6.6	5.8	6.0	5.6	5.8
90	8.2	8.2	8.2	8.1	7.0	6.4	7.0	6.5
100	8.8	5.5	7.8	7.6	7.0	4.2	7.0	6.9
**220–300 GHz band**								
240	7.6	—	0.0	7.0	5.4	—	0.6	5.0
270	6.4	—	2.0	6.4	6.5	—	4.6	6.3
300	7.0	—	3.7	6.7	6.4	—	3.0	6.0
